# International Xenotransplantation Association (IXA) Position Paper on the History, Current Status, and Regulation of Xenotransplantation

**DOI:** 10.1097/TP.0000000000005373

**Published:** 2025-04-08

**Authors:** Wayne J. Hawthorne, Richard N. Pierson, Leo Buhler, Peter J. Cowan, Jay Fishman, Rita Bottino, Raphael P. H. Meier, Paolo Brenner, Eckhard Wolf, Emanuele Cozzi, Muhammad M. Mohiuddin

**Affiliations:** 1 The Centre for Transplant & Renal Research, Westmead Institute for Medical Research, Westmead, NSW, Australia.; 2 Department of Surgery, School of Medical Sciences, University of Sydney, Westmead Hospital, Westmead, NSW, Australia.; 3 Center for Transplantation Sciences and Division of Cardiac Surgery, Department of Surgery, Massachusetts General Hospital and Harvard Medical School, Boston, MA.; 4 Cantonal Hospital Fribourg, Faculty of Science and Medicine, University of Fribourg, Fribourg, Switzerland.; 5 Immunology Research Centre, St. Vincent’s Hospital Melbourne, and Department of Medicine, University of Melbourne, Melbourne, VIC, Australia.; 6 Transplant Infectious Diseases and Compromised Host Program, Massachusetts General Hospital and Harvard Medical School, Boston, MA.; 7 Imagine Pharma, Pittsburgh, PA.; 8 Department of Surgery, University of Maryland School of Medicine, Baltimore, MD.; 9 Department of Cardiac Surgery, University Hospital, LMU Munich, Munich, Germany.; 10 Gene Center, LMU Munich, Munich, Germany.; 11 Transplant Immunology Unit, University Hospital of Padua, Padua, Italy.; 12 Cardiac Xenotransplantation Program, University of Maryland School of Medicine, Baltimore, MD.; Sichuan Provincial People’s Hospital, Chengdu, Sichuan, China; Transplant Research and Xenotransplantation Laboratory, Indiana University, Indiana, USA; College of Veterinary Medicine, Yunnan Agricultural University, China; University of Alberta, Canada; Swiss National Science Foundation project “Xeno2Cure”, Switzerland; Konkuk University Medical Center, South Korea

**Keywords:** genetic engineering, guidance, legislation, organ donation, regulations, transgenes, xenotransplantation, xenozoonoses

## Abstract

Recent landmark clinical translation of xenotransplantation depended upon multiple innovations by the xenotransplant community, including the introduction of a variety of source pig genetic modifications, technical innovations, and novel immunosuppressive strategies, as well as the development of ethical and regulatory frameworks to support translation to the clinic. Each organ, tissue, or cell type intended for xenotransplantation will require application-specific preclinical milestones to be met in order to predict “success”, as measured by ethical, safe, and efficacious translation to the clinic. Based on successful pre-clinical results and emerging evidence from decedent studies and initial clinical cases, evidence-based infectious disease, ethical, and regulatory considerations are emerging, and will be the foundations for the application-specific position papers that are currently under development. Here, we describe significant landmark events focusing upon safe and efficacious results underpinned by appropriate guidance documents developed over the past three decades that enabled recent translation to the clinic for heart and kidney xenografts. These steps have been undertaken over the past three decades by the xenotransplant community specifically led by the International Xenotransplantation Association (IXA) in consultation with the Transplantation Society (TTS) and the World Health Organization (WHO) to usher xenotransplantation to the clinic.

## 1 | INTRODUCTION

Due to the ever-increasing gap between supply and demand for transplantable human organs for transplantation, over the past three decades, the xenotransplantation community has focused its research efforts on developing a viable alternative to allotransplantation [1, 2]. Scientific investigation has been undertaken in parallel with the development of guiding ethical principles and evidence-based regulatory oversight as the basis for building a logical, safe, and efficacious path to the clinic [3]. Both scientific research and regulatory guidance efforts have been focused on the goal of developing a readily available, predictably efficacious, affordable technology for worldwide application [4, 5].

Scientific advances have occurred on a number of fronts as the result of a concerted effort primarily led by members of the International Xenotransplantation Association (IXA) to usher xenotransplantation to the clinic. Pre-clinical testing in non-human primate (NHP) models has sought to define the most appropriate genetic characteristics of source animals for each proposed xeno organ, tissue, or cell type, and in some instances required development or adoption of supportive technology platforms (e.g., ex vivo heart perfusion for ischemia minimization) [6, 7]. In addition, increasingly effective immunosuppressive protocols and herd and donor health screening protocols have been developed [8], piggery practices refined, and animal facilities built to enable efficient production of “designated pathogen free” animals and organs [9]. In parallel, IXA, in consultation with the Transplantation Society (TTS) and the World Health Organization (WHO), has contributed to the development of the appropriate regulatory guidances and standards around the globe for more than 25 years [10]. The journey undertaken over this time has not been without hurdles. For example, in the 1990s the risk that pandemic infection (“xenozoonosis”) might occur as the result of xenotransplants from NHPs or pigs resulted in government moratoria preventing clinical xenotransplant trials. Here, we describe the positive advances that have enabled and justified advancing to the clinic. The purpose of this IXA document is to frame a series of planned organ-, tissue-, and cell-type-specific “Commentaries” that are focused on the particular aspects required to achieve successful clinical xenotransplantation for each planned application.

## 2 | BACKGROUND

Contemporary clinical xenotransplantation commenced in the 1960s with early pioneers utilizing NHPs as donors, between 1963 and 1964 Reemtsma et al. performed six xenotransplants using chimpanzee kidneys [11]. At a similar time, Starzl performed six baboon kidney xenotransplants [12], and he also explored the possibility of performing liver xenotransplants. In 1970, Starzl and his team successfully performed a liver xenotransplant from a chimpanzee into a child [13]. Possibly the most controversial of these early xenotransplants were those of the cardiac xenotransplants with Hardy et al. performing the first chimpanzee-to-human heart transplant in 1964 [14]. Due to both public and medical community scrutiny of this transplant, no further cardiac transplants were performed for a decade until Barnard et al. performed two heart xenotransplants in 1977, one from a baboon and the other from a chimpanzee [15]. By far the best known clinical cardiac xenotransplant was that performed by Leonard Bailey, who in 1984, successfully transplanted a baboon heart into an infant known as “Baby Fae”. The infant survived for 20 days but the graft was ultimately lost from rejection [16]. Once again this drew significant ethical scrutiny from the community for the use of NHP as organ donors [17].

Due to these ethical quandaries and the ideas that NHP could never viably be adapted as organ donors, several groups performed exploratory clinical cardiac xenotransplants from other animals. The first recorded pig heart xenotransplant was performed in London in 1968 by Ross and coworkers, which unfortunately was the first clinical cardiac xenotransplant to undergo hyper acute rejection (HAR) [18]. Around the same time in 1968, Cooley et al. performed an orthotopic sheep-to-human cardiac xenotransplant at the Texas Heart Institute in Houston which also resulted in immediate graft loss from HAR [19]. Other less publicized attempts occurred over the next few years with little if any success, due to preformed xenoantibodies and the lack of suitable immunosuppression.

Decades later, it was thought that the newer immunosuppressive agents could help prevent the overwhelming xenograft rejection, and a number of clinical xenotransplants were performed with limited insight into the immunological processes involved. In 1992, Religa and coworkers performed a pig heart xenotransplant at the Sosnowiec Cardiac Institute, Poland [20]. Even more controversially in 1997 Dhani Ram Baruah undertook a pig heart xenotransplant at his Heart Institute in India. With the patient tragically dying, Baruah was subsequently arrested for violation of the country’s Human Organ Transplantation Act of 1994 [21].

However, there were several more controlled studies following decades of pre-clinical research in Sweden by Carl Groth who led a study to treat diabetic patients during 1992 using porcine fetal islet xenografts and pig kidney cross-circulation in patients with renal failure [22]. And later in 1994, Makowa et al. in Los Angeles, USA successfully used a pig liver as a bridging graft for a patient suffering fulminant hepatic failure [23].

However, these efforts were halted in the 1990s for several reasons. Principally, ethical concerns arose concerning the use of endangered species of NHPs such as chimpanzees, with their limited availability, human-like social networks, and other behaviors. Further, in the early AIDS era, research demonstrated that primate endogenous retroviruses (ERVs) may infect human cells, potentially leading to widespread xenozoonotic infections in humans [24]. Similarly, porcine ERVs (PERVs) were found to infect human cell lines [25]. As a result, a moratorium on xenotransplantation was implemented in countries such as the USA, Australia, UK, and several other jurisdictions [26].

However, there were some xenotransplant activities still being undertaken with inadequate regulatory oversight. These included in the 1990s encapsulated porcine islet transplants implanted into adolescents in Mexico where there were no guidelines nor regulations in relation to xenotransplantation [27]. Along with another unregulated program in the 1990s and early 2000s, in Moscow, Russia where there were no regulations on xenotransplantation. Apparently, Shumakov and coworkers transplanted hundreds of diabetic patients with islet cells obtained from rabbit pancreata. However, the reports on these studies are very incomplete and no information on the outcome of this trial is available. Additionally, transplantation of fetal pancreatic tissue from pigs and calves has also been performed to significant numbers of patients in China but few results have been reported internationally [28]. One of the few internationally published studies from China was from Wei Wang where they performed porcine islet transplants into diabetic patients [29]. These ongoing cases prompted TTS and IXA, in collaboration with the WHO, to increase efforts to establish effective worldwide oversight of and guidance regarding safe, scientifically sound clinical xenotransplantation practices [30].

## 3 | ORIGINS OF IXA

**FIGURE 1.**
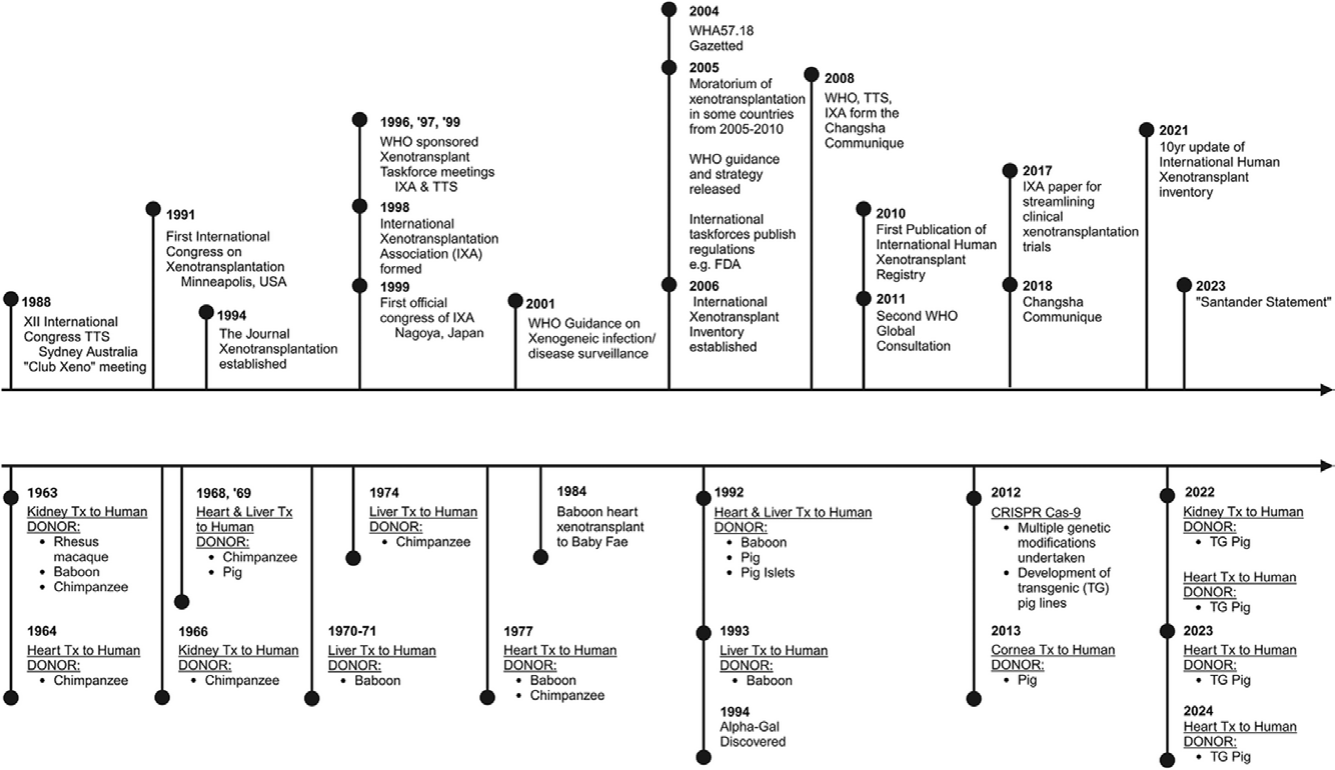
Timeline of the development of major xenotransplant practice and oversight development.

As interest in developing clinical xenotransplantation emerged in the 1990s, the leaders of this field instigated a series of meetings, starting at Arden House by those initial pioneers of the field (Reemtsma, Hardy, 1989), and continuing in Minneapolis (Najarian, Platt, Dalmasso, Cooper, 1991), Cambridge (1993, White, Wallwork), Boston (Bach, 1995), and Nantes (1997, Soulillou) to update the WHO on progress in xenotransplantation research. As can be seen in the timeline of major events in the field of xenotransplantation (Figure 1), this culminated in the official formation of the IXA, which was incorporated as the first official “section” of TTS [31] at the XVII International Congress of TTS in Montreal in 1998, and held its first meeting the following year in Nagoya (1999).

## 4 | DEVELOPMENT OF XENOTRANSPLANTATION GUIDANCE DOCUMENTS

Pivotal to the development of guidance documents for the field, the WHO sponsored a series of Xenotransplantation Task Force meetings arranged by the WHO, Division of Emerging and Other Communicable Diseases and held in 1996, 1997 and 1999. They worked with the IXA, TTS and international experts to develop international guidance on clinical xenotransplantation with respect to safety, equitable access, and standardization of regulations [32].

However, the overarching concern that clinical xenotransplantation may carry public health risks when xenotransplanted organs, tissues, or cells were transplanted into heavily immunosuppressed patients led to the first guidance document to be published with the title “WHO guidance on xenogeneic infection/disease surveillance and response: a strategy for international cooperation and coordination 2001” [33]. Following the release of this guidance, several international jurisdictions developed taskforces and published regulations in relation to xenotransplantation, the first country being the USA, where the Food and Drug Administration (FDA) produced a draft guidance that same year [34].

Even with the publishing of the WHO guidance on xenogeneic infection/disease surveillance and response and additional ongoing efforts by the WHO, TTS, and IXA, international disease surveillance and response systems were still developing and lacked consistency between WHO member states. This vulnerability was highlighted by the SARS-CoV-1 outbreak of 2002–2003 [35]. Xenotransplantation experts emphasized the need for advanced health infrastructure, diagnostic technology, and regulatory frameworks, which were largely absent or inadequate in member states at that time [36]. As a consequence, strategies to minimize risk were established [37, 38]. In particular, categories of potential human pathogens that could emerge from bird, insect, and other animal reservoirs were developed and published, with examples of validated microbiological assays [9]. For the specific instance of xenotransplantation activities, lists were compiled by the IXA infectious diseases task force [37]. National governmental organizations including the FDA, OECD [36], and the Infection Surveillance Steering Group of the UK Xenotransplantation Interim Regulatory Authority (UKXIRA) established a series of recommendations, highlighting the importance of establishing criteria for source pig characterization and “designated pathogen free” status, and explicit guidelines for xenotransplantation clinical trial conduct, including recipient monitoring criteria and recommendations regarding regulatory oversight [39, 40].

The IXA and TTS, under the auspices of the WHO, built on the framework of the “WHO Guidance on Xenogeneic Infection/Disease Surveillance and Response” document [33] by holding the first WHO consultation on transplantation ethics, access, and safety in Madrid in October 2003, with experts from 23 countries. This led to the World Health Assembly Resolution WHA57.18 in 2004, which encouraged member states to permit xenotransplantation trials only when effective national regulatory controls were in place, and called for establishing global data collection on xenotransplantation practices [41].

Following publication of WHA57.18, international bodies including the FDA, UKXIRA, SACX, European Medicines Agency (EMA), and the Australian Xenotransplantation Working Party reviewed and acted upon the WHO advisory recommendations, resulting in new laws and/or regulations in each of these jurisdictions. In 2003, IXA’s Ethics Committee published its inaugural “position paper” that emphasized the importance of peer-reviewed preclinical data as the necessary basis for the design and conduct of a clinical trial, including the informed consent process, and the ethical necessity for effective oversight of clinical xenotransplantation research by competent authorities as a precondition for regulatory approval [42]. In this landmark document, five potential advantages of xenotransplantation were articulated to justify the risks and costs associated with xenotransplantation activities:

1. In countries where human organ donation has not been accepted for ethical or cultural reasons, xenotransplantation might provide an acceptable alternative.
2. In several respects, xenogeneic organs would offer advantages similar to those associated with the use of human live donor organs—the transplant procedure can be scheduled; recipient pre-treatment is feasible; the quality of the organs will be known in detail; there will be minimal warm and cold ischemia times; and the influence of the various pathophysiologic consequences of brain death on organ quality will be avoided.
3. With ready access to organs, recipient selection criteria could be broadened.
4. Xenogeneic transplants might not be susceptible to the human autoimmune diseases or viral infections that caused organ failure in the first place, and which often limit the survival of allogeneic organ transplants.
5. The use of inbred, immunologically standardized source animals would facilitate pretransplant tolerance induction. Source tissue could also be modified by genetic engineering to minimize graft rejection, optimize physiological function, and provide other potential advantages to the recipient.

Significant work was also undertaken by the Committee of Ministers of the Council of Europe (CoE) which set up a Working Party on Xenotransplantation in 1999 under the joint responsibility of the Steering Committee on Bioethics (CDBI) and the European Health Committee (CDSP). In 2003 the CoE published their report on the state of the art in the field of xenotransplantation [28]. However, it was not until 2006 that EMA published the guidelines under the EMEA/CHMP/410869/2006 [43] which is appended with the EMEA/CHMP Points to consider on Xenogeneic Cell Therapy Medicinal Products (CPMP/1199/02) [44]. An updated annex to the guideline on xenogeneic cell-based medicinal products (EMEA/CHMP/CPWP/83508/2009) deals more specifically with requirements for xenogeneic products [45].

Following these landmark documents, the WHO, in conjunction with the IXA and TTS, directed greater attention to xenotransplantation, jointly convening the first WHO Xenotransplantation Advisory Consultation in Geneva at the WHO headquarters in 2005. A major focus of this meeting was to address the inadequacies in global organ donation rates and to recognize the valuable role that xenotransplantation could play in alleviating this problem [30]. The consultation underscored the need for effective regulatory systems, including patient tracking and long-term archiving of source animal and recipient blood and tissues.

## 5 | DOCUMENTING CLINICAL XENOTRANSPLANTATION

Increased awareness regarding the existence of unregulated or under-regulated xenotransplantation activities in multiple jurisdictions led to the establishment of a global inventory of xenotransplantation practices (www.humanxenotransplant.org) [46]. Established with support from IXA, the University Hospital Geneva, and the WHO, the inventory of xenotransplantation practices went live in October 2006 and published its first report in 2010 in the journal *Transplantation* [47]. It provides information on the clinical applications of xenotransplantation since 1995 and includes data gleaned from regular (monthly) internet searches along with review of scientific journals, congress, and IXA manuscript or abstract submissions. Since its inception, the registry has documented 54 clinical xenotransplantation protocols performed on humans. A full review of these activities is published [48] and is briefly summarized below in the following section.

For the period from 1995 to 2010, 29 clinical xenotransplantation study protocol applications were recorded. These involved xenogeneic cells (e.g., pancreatic islets, kidney, chromaffin, stem, and fetal or adult cells/cell products) and extracorporeal perfusion (e.g., hepatocytes in a bioreactor/filter, or whole liver, spleen, or kidney organ perfusions). These procedures occurred in 12 countries, nine of which lacked xenotransplant-specific national regulations that were in concordance with those of the WHO guidelines and the “Changsha Communiqué 2008” [49]. This information was used by IXA leadership to leverage the support of the TTS and WHO to inform national health authorities, healthcare staff, and the public. It also provided the impetus for the review of the “Changsha Communiqué” and the publication of the 2011 update [50]. Investigators and their associated health care regulatory authorities were encouraged to adopt WHO-recommended internationally harmonized guidelines and best practices for regulation of xenotransplantation. Efforts by IXA, TTS, and the WHO have led to the development of policies and regulations in most jurisdictions [47].

In 2021, the registry published a 10-year follow-up covering activities between 2010 and 2020 [46]. Clinical xenotransplantation trials performed between 2010 and 2020 all used pigs as the organ, tissue, or cell source. These studies included trials of islet cells to treat diabetes, 1 trial, undertaken in 14 patients in New Zealand and 8 patients in Argentina receiving encapsulated islet cells, another trial was undertaken in China of neonatal islet cells into 32 patients. Porcine skin grafts were used to treat burn patients, the trial was undertaken in the USA on six patients. Another trial undertaken in China of the acellular cornea was used to treat 25 patients suffering from fungal keratitis. Another trial was undertaken in New Zealand by Elliot and coworkers where they treated 12 patients suffering from Parkinson’s disease, they used encapsulated neonatal porcine choroid plexus cells implanted into the putamen. Of major importance to note is that all procedures were undertaken with regulatory approval [46].

An interval analysis in May 2024 finds a recent upsurge of clinical applications of solid organ xenotransplants both using brain-dead “decedents” as a “‘preclinical model” (two hearts, seven kidneys), and in two heart, two kidney, and one auxiliary liver xenotransplant in patients treated with therapeutic intent [48].

## 6 | BRIEF SUMMARY OF CLINICAL XENOTRANSPLANTATION TRIALS

As recorded by the International Xenotransplantation Inventory and overviewed above, clinical xenotransplantation trials have involved various tissues and organs. A few select examples of these trials include:

- *Pancreatic islet cells*: Wang et al. (2011) reported on the xenotransplantation of neonatal porcine islets in 22 patients with type 1 diabetes, observing reduced insulin requirements and no serious adverse events or evidence of porcine endogenous retrovirus (PERV) transmission [29]. A 2017 update from Wang’s team reported at a press conference in 2017 that 10 more patients who received xenoislets during 2013–2017 had a reduction in insulin needs by over 60% and significant patient improvement. Matsumoto et al. (2014) studied encapsulated porcine islets in 14 type 1 diabetes patients without immunosuppressive therapy, noting decreased hypoglycemic events but no impact on HbA1c levels [51]. A subsequent 2016 study by Matsumoto and coworkers in Argentina, involving eight patients followed for up to 600 days, showed improved HbA1c levels and no PERV infection [52].
- *Choroid plexus cells*: In 2013, Living Cell Technologies transplanted encapsulated neonatal porcine choroid plexus cells into 12 patients with Parkinson’s disease. The study found few if any patients demonstrated any change to their Parkinson’s [53–55].
- *Skin*: In 2019, researchers at Massachusetts General Hospital performed a Phase 1 clinical trial transplanting genetically engineered (GE) porcine skin onto wounds in six burn patients. The porcine skin functioned similarly to human cadaveric skin as a wound dressing, with no adverse effects reported [56, 57].
- *Cornea*: Chen et al. (2019) conducted a study from 2013 to 2014 using acellular porcine corneal stroma in 25 patients with fungal keratitis. They reported transparent grafts in 20 patients at 1-year follow-up [58].
- *Kidney*: In September and November 2021, NYU Langone Health performed xenotransplants of thymo-kidneys from *α*1,3-galactosyltransferase gene-knockout pigs into brain-dead patients. The kidneys functioned immediately, with no evidence of hyperacute rejection (HAR) despite a positive crossmatch in one recipient [59]. In early 2022, the University of Alabama Birmingham transplanted two GE porcine kidneys into a brain-dead patient. The kidneys functioned and produced urine without immediate rejection, although there was minor thrombotic microangiopathy and variable urine output [60]. In the next year, the same group also performed a brain-dead adult decedent who underwent bilateral native nephrectomies followed by 10 gene-edited (four gene knockouts, six human transgenes) pig-to-human xenotransplantation. Physiologic parameters and laboratory values were measured for 7 days in a critical care setting and demonstrated these kidneys provided physiologic balance until elective study termination [61].
- In a world-first procedure, the Harvard Medical School team at Massachusetts General Hospital transplanted a genetically edited pig kidney into a 62-year-old living patient in March 2024. This innovative surgery utilized a genetically modified (GM) pig kidney with 69 genetic edits designed to reduce the risk of organ rejection and improve safety and compatibility with human tissues. The patient, who had end-stage renal disease, experienced initial success, with the transplanted kidney functioning well until the recipient’s death, presumably caused by a cardiac arrhythmia, 52 days following implantation. Metagenomic testing did not reveal any infection due to porcine microorganisms in the recipient. This procedure not only highlighted the potential of xenotransplantation in addressing the global organ shortage but also provided valuable insights into immune responses and encouraging evidence that at least intermediate-term viability (months to years) is likely, marking a significant advance in kidney xenotransplant medicine. In 2024, New York University Langone Health performed a procedure involving a mechanical heart pump and a gene-edited pig kidney. The patient received the pig kidney along with a pig thymic tissue to prevent rejection. Unfortunately, after 6 weeks, the kidney failed due to recipient hypotension, necessitating its removal. A comprehensive summary of these cases is provided in the accompanying manuscript—IXA Position Paper on Kidney Xenotransplantation.
- *Heart*: On January 7, 2022, the University of Maryland performed the first transgenic pig-to-human cardiac xenotransplant in a patient with non-ischemic heart disease maintained on extracorporeal membrane oxygenation (ECMO) support and being immobile for 60 days in the hospital. The procedure, permitted under expanded access by the FDA, used a 10-gene modified pig and an anti-CD40-based immunosuppression regimen. The heart functioned well for 49 days before developing cardiac dysfunction associated with the activation of porcine CMV in the heart xenograft, along with the detection of rising anti-pig antibody levels in the recipient [62]. Based on the learning from the first transplant, the University of Maryland’s second transgenic pig-to-human cardiac xenotransplant is also under the “expanded access” rubric. It was conducted in September 2023, once again marking a significant advancement in medical science. The procedure again involved implanting a 10-transgene pig heart into a 58-year-old patient with terminal heart failure, in this case under treatment with an anti-CD154-based immunosuppressive regimen. The patient exhibited excellent initial heart function through several serious post-operative complications. However, after a few weeks, graft function declined in association with rising anti-pig antibody titers that proved refractory to augmented immunosuppressive treatments, leading to the patient’s passing 40 days post-transplant. These cases clearly demonstrated life-supporting pig heart function in both patients; however, based on the presumption that anti-pig immune responses by both patients may have contributed to their demise, identified the need for better immunosuppressive strategies. These clinical pilot studies, supported by cutting-edge molecular diagnostics, significantly advanced the field’s understanding and paved the way for future clinical trials.

In summary, to date the major initial obstacle to pig-to-human xenotransplantation, “hyperacute rejection”, has been avoided in all of the decedent and living recipients reported to date. Pro-longed life-supporting xenograft function has been demonstrated for both multi-GE pig hearts and for GalTKO and multi-GE pig kidneys. These pilot studies, along with future preclinical studies and additional clinical efforts, will together inform the design of formal IND-qualifying clinical trials on carefully selected patients. Patient selection and source pig genetics to be used will likely differ for various organs, tissues, and cells; these details will be outlined in the specific “White Papers” for each disease/organ categories, to be published as companion papers to this “Overview”.

## 7 | THE CHANGSHA COMMUNIQUé AND ANNEXES

Motivated by persistent disparities in xenotransplantation regulatory posture among WHO member states, the first WHO Global Consultation on Regulatory Requirements for Xenotransplantation Clinical Trials was held in Changsha, China in 2008 under the leadership of WHO’s Dr. Luc Noel [49]. The resulting Changsha Communiqué broadly inscribed 10 guiding principles and 20 recommendations for consideration by “the WHO, Member States, Investigators, and proposers of clinical trials using xenotransplantation products”. This pivotal document provided evidence-based guidance as well as expert opinion regarding clinical trial design and was a landmark document in establishing guidance for xenotransplantation. It also strengthened the collaborative link between the IXA and TTS and the WHO. This guidance was approved by the WHA and published in 2009 [49, 63]. The guiding principles addressed the ethical and safety considerations surrounding xenotransplantation, emphasized the need for rigorous scientific research, robust ethical standards, and the protection of public health and animal welfare, and advocated for transparent risk assessment, informed consent, and international collaboration to minimize potential risks and address ethical concerns. The recommendations provide detailed specific guidance on ensuring the safety and efficacy of xenotransplantation practices, including the establishment of regulatory frameworks, the implementation of stringent biosafety measures, and the promotion of public and scientific dialogue to ensure responsible and ethical advancements in this field.

In 2011, the WHO and IXA organized the Second WHO Global Consultation on Regulatory Requirements for Xenotransplantation Clinical Trials, held at the WHO headquarters in Geneva [50]. This meeting focused on the refinement of guidance for infectious disease surveillance, prevention, and how best to respond appropriately to various clinical xenotransplant trial scenarios [50] significantly updating the 2001 document (WHO Guidance document on Xenogeneic Infection/Disease Surveillance and Response: A strategy for International Cooperation and Coordination) [24, 33, 50].

As a direct outcome of the 2011 meeting, IXA infectious disease experts and other participants from the Consultation published a position paper in 2012 on xenotransplantation-associated infectious risks and proposed several preventative and mitigation strategies [24]. These importantly suggest that infectious disease events linked to clinical xenotransplantation from swine are likely to be rare, and current human trials have not shown transmission of porcine microorganisms, including porcine endogenous retrovirus (PERV). This document outlined disease surveillance strategies for recipients of non-human tissues, emphasizing that infection risk depends on the pathogen’s characteristics, transmission quantity, host factors, and immune competence. Predicting pathogen behavior in human hosts is challenging and investigations into suspected xenogeneic infections should involve expert review panels and public health authorities, especially for immunocompromised patients. Reporting outcomes, including infections, is crucial for scientific transparency and patient care. Safe xenotransplantation will require surveillance for both known and novel pathogens, validated microbiological assays, and long-term sample repositories. International collaboration and secure data sharing are essential, and public engagement about potential infectious risks is important due to recent concerns such as the recent COVID pandemic [24].

In 2017 the IXA council and xenotransplant experts published a paper focusing on streamlining the approaches to regulation of clinical xenotransplantation trials [64]. This article strongly urged the national regulatory authorities worldwide to re-examine their guidelines and regulations regarding xenotransplantation. This paper urged for better design and conduct of safe and informative clinical trials of cell and organ xenotransplantation, when and as supported by preclinical data [64]. This paper was one of the impetuses to update the Changsha Communiqué.

On the 10th anniversary of the initial Changsha meeting, IXA and TTS, with WHO assent along with FDA, EMA, and the Chinese FDA participation, reconvened subject matter experts in Changsha in December 2018 to review and update previous recommendations from guidance and position statements in light of scientific and technological developments. More than 70 revisions to the previous Communique were recommended and published under the aegis of the IXA council [63] ensuring that the original recommendations to WHO were preserved. The emphasis remained to promote public awareness of xenotransplantation, of both the potential benefits, as well as precautions, and the need for effective internationally consistent regulation by member states.

## 8 | IXA COUNCIL RECOMMENDATIONS FOR ACTIONS AND STRATEGIC OBJECTIVES

### 8.1 | Undertaking Public Consultation

As stated in the latest Changsha Communiqué, public engagement and consultation are important to build societal acceptance of a new treatment that exposes the world’s population to new risks, including the remote risk that xenotransplantation could expose humankind to the emergence of a new pandemic infection [63].

Until now, public engagement regarding xenotransplantation trials has not been systematically undertaken. Active research in the field of xenotransplantation in the 1990s/2000s was associated with several reports related to the ethical and safety aspects of xenotransplantation [65, 66]. Overall, these were favorable, with support of xenotransplantation by the major religions based on easing the suffering of patients if the basic principles of biomedical ethics are observed and animals are treated humanely and with respect [65, 67, 68].

A common assumption is that patients with end-stage organ failure who are not eligible for allotransplantation will be enthusiastic participants in xenotransplantation programs and several studies have demonstrated this [3]. However, this has not always been the case, as one study reported that a proportion of acutecare nurses were opposed to xenotransplantation, although this was in the late 1980s when primates were the donors of choice for clinical xenotransplantation [69]. This negative perception has changed as there seems to be less issue with the pig as the donor source animal. However, concerns raised include animal rights and welfare, patient acceptance and informed consent, risk of infectious diseases, and broader public health issues [70].

The costs of organ transplantation underlie the potential for inappropriate or corrupt practices in living donor or even cadaveric organ donation in poorer socioeconomic countries. The gap between demand and supply of organs for transplant has led to organ trafficking, organ tourism, and commercialism. This problem remains an overwhelmingly negative issue and raises ethical dilemmas that could be resolved if xenotransplantation were successful and broadly adopted [5, 71]. Additionally, multiorgan donation costs are extensive, complicating current financial repatriation to the donor family, donor hospitals, organ donor agencies, and all involved in the extensive logistics of the organ donor process. The ability to acquire a GE pig organ whenever required through a simple commercial transaction (as in the purchase of a life-saving drug) will be of great benefit to the patient and society [72].

In today’s information-driven society, public consultation is crucial for ensuring that individuals are well-informed and able to make educated decisions about complex issues such as xenotransplantation. As advancements in medical science continue to push boundaries, the public demands comprehensive and transparent information to understand the implications of new technologies. Effective public consultation involves not only providing detailed background information but also engaging in open dialogues that address the diverse perspectives and concerns of the community. This approach ensures that decisions are made with a thorough understanding of both the potential benefits and risks, fostering a more informed and participatory society.

There are several avenues by which we can move forward, but the IXA leadership believes that a systematic public consultative process performed at an international level should be undertaken as the basis for developing a consensus on the multiple challenging aspects from both safety and ethical areas associated with xenotransplantation clinical translation. The IXA is concerned that if clinical trials take place without proper consultation of health professionals and the public at large, there is a risk that xenotransplant activities could generate a negative perception among the public, which could degenerate rapidly via social media into a world-wide negative perception of the field, likely delaying or preventing evaluation of potentially life-saving new xeno-based treatments. In the estimation of IXA Council, it is important to be pro-active and to proceed deliberately in anticipation of clinical application of xenografts, rather than to act in response to a crisis, trying to deal with foreseeable concerns after the fact. In Table 1. we propose a plan for public consultation on xenotransplantation for clinical application. This plan aims to educate, foster transparency, build trust, and ensure that the public’s voice is heard and considered in the decision-making process related to xenotransplantation.

**TABLE 1:** Approaches to the development of public consultation in xenotransplantation.

Phase	Step	Description
1. Preparation phase	1.1 Define objectives	- Clearly articulate the goals of the public consultation.
		- Identify what you hope to achieve (e.g., gather public opinions, educate the community, and identify concerns).
	1.2 Stakeholder identification	- Identify key stakeholders, including patients, healthcare professionals, ethicists, animal rights groups, and the general public.
		- Consider forming an advisory panel with representatives from diverse groups.
	1.3 Develop educational materials	- Create comprehensive, accessible materials that explain xenotransplantation, its potential benefits, risks, and ethical considerations.—Use multiple formats (brochures, videos, online content) to reach a broad audience.
2. Engagement phase	2.1 Launch informational campaign	- Utilize various media channels (social media, news outlets, community bulletins) to inform the public about the consultation. - Ensure materials are accessible in different languages and formats to reach diverse populations.
	2.2 Host public forums and workshops	- Organize town hall meetings, webinars, and workshops to provide information and facilitate discussions.
		- Include expert speakers, interactive Q&A sessions, and opportunities for small group discussions.
	2.3 Online consultation platform	- Develop an online platform where people can submit questions, comments, and feedback.
		- Provide resources and updates regularly to keep participants informed.
3. Feedback collection	3.1 Surveys and polls	- Distribute surveys and polls to gather quantitative data on public opinions and concerns.
		- Ensure questions are clear and unbiased to obtain accurate feedback.
	3.2 Focus groups	- Conduct focus groups with diverse segments of the population to explore opinions in greater depth.
		- Include a mix of stakeholders to ensure a broad range of perspectives.
4. Analysis and reporting	4.1 Data analysis	- Analyze the feedback collected from surveys, focus groups, and public forums.
		- Identify key themes, concerns, and recommendations.
	4.2 Reporting	- Prepare a comprehensive report summarizing the findings, including public concerns, suggestions, and general sentiment.
		- Present the report in an accessible format to stakeholders and the public.
5. Implementation and follow-up	5.1 Policy recommendations	- Based on the consultation findings, develop or adjust policies and guidelines related to xenotransplantation.
		- Ensure recommendations address the concerns and preferences expressed by the public.
	5.2 Ongoing engagement	- Establish mechanisms for continued public engagement and feedback as xenotransplantation research and practices evolve.
		- Provide regular updates on how public input is being incorporated into decision-making processes.
	5.3 Evaluation	- Assess the effectiveness of the consultation process and make improvements for future engagements.
		- Seek feedback on the consultation process itself to refine methods and strategies for further consultation and implementation.

### 8.2 | Refining Regulatory Guidance for Xenotransplantation

For xenotransplantation to be equitably and safely introduced into worldwide clinical practice, robust long-term monitoring of xenotransplantation activities and outcomes must be established, consistent with prior WHO guidance and current IXA recommendations [63].

Refinements and updates to current WHO guidelines and regulatory guidance are recommended by the IXA council based upon the 2018 Changsha Communiqué. This is clearly in line with the ongoing technological advancements and findings from recent studies now entering the clinic with a focus to ensure the most up to date implementation of guidance at a global level.

Representatives from all disciplines should continue to be involved to discuss progress and innovations in the field, to mirror this progress with respect to the provision of guidelines to provide member states with the guidance to provide objective and informed regulatory oversight, and to foster this process at the global level under the umbrella of the three organizations: IXA, TTS, and WHO. The IXA council has submitted a proposal to TTS and WHO that the next update of WHO guidance on xenotransplantation should take place in Geneva in 2025 around the time of the IXA congress. This update will focus once again on the significant advances underway in the field of xenotransplantation, including further development of geneedited source animals, screening technologies to ensure best practice with the most up-to-date techniques, and appropriate choice of source pig genetics for the various organs, tissues, or cells to be transplanted. There should be a strong emphasis on appropriate education, consultation, and discussion around the application of clinical xenotransplantation.

### 8.3 | Xenotransplantation is Integral to Ethical Standards for Clinical Transplantation

A Global Summit was held in November 2023 in Santander, by the Organización Nacional de Trasplantes (ONT), under Spain’s EU Council Presidency. The Summit was titled “Towards Global Convergence in Transplantation: Sufficiency, Transparency and Oversight” and proposed a global agenda for transplantation over the next decade. It was co-organized by the European Directorate for the Quality of Medicines and Healthcare (EDQM) of the Council of Europe, the European Society for Organ Transplantation (ESOT), and TTS, and co-sponsored by WHO and supported by the International Society of Nephrology (ISN). The Summit reviewed current transplant practices, identified existing limits to access and obstacles to achieving optimal outcomes with available organs, and sought to inform future scientific directions, regulatory initiatives, and legal efforts around transplant activities to benefit people worldwide. The event, attended by 183 participants from 57 countries, produced recommendations for national and regional governments, published as the “Santander Statement” [73]. Because xenotransplantation is perceived to be an important component of future transplant activities, representatives from the IXA were invited to participate.

The motivating principle behind the Santander 2023 meeting was to universally improve care of patients requiring organ replacement by various means.

However, we can see that these can also support the role of xenotransplantation by “prioritizing transplant therapy when that is the most cost-beneficial treatment modality for patients”; and to “supporting low-resource countries in developing and strengthening their transplant systems”. Also to offer patients an option that is not available through conventional transplants [73, 74]. The issues remain how best to triage patients between allo- and xenotransplantation. Also how to do so differently for countries that currently do not have robust transplant systems and with limited if any transplant access.

To provide such advice and develop appropriate guidance documents, the IXA proposes that an updated set of guidelines should involve a multi-faceted approach that integrates scientific, ethical, and regulatory perspectives. Firstly, a comprehensive review of existing guidelines from prominent health organizations, led by the WHO and the IXA, provides a foundation for scientific, regulatory, and ethical considerations [63]. This includes evaluating safety, efficacy, and long-term outcomes to address potential risks such as zoonotic infections and immunological responses. Secondly, engaging a diverse group of stakeholders, including bioethicists, clinicians, patients, and regulatory bodies, ensures that multiple viewpoints are considered [75]. Public consultations and generally accepted existing ethical frameworks, like the Declaration of Helsinki and the Belmont Report, can guide the development of standards that respect human rights and social values; xeno-specific features need also to be considered [76]. Finally, harmonizing regulations across countries, facilitated by international collaborations and agreements, is highly desirable for the consistent application of ethical standards and to address cross-border ethical dilemmas, and we suggest that this can best be done if WHO and member states agree that all xeno trials should be undertaken with the collaboration, engagement and support of the IXA, TTS and the WHO. The ideal setting for this undertaking is by comprehensively reviewing the current guidelines for xenotransplantation by international experts in an update of the Changsha Communiqué.

### 8.4 | Educating the Global Medical Community Regarding Xenotransplantation

IXA identifies an urgent need for the global health community to comprehensively address the multiple ethical issues associated with xenotransplantation clinical trial design and conduct. In partnership with TTS, IXA leadership proposes to facilitate further engagement with subject experts from various fields of medicine to help draft guidelines for trial design and conduct, and to inform related regulatory decision-making. The IXA considers that it would be very timely to address such issues in advance of any formal clinical trial announcement; IXA proposes to develop a formal program for a systematic official WHO-sanctioned consultation process regarding the Ethics of Xenotransplantation with the support of TTS and WHO.

### 8.5 | Scientific, Ethical, and Regulatory Oversight of Clinical Xenotransplantation Trials

The IXA recommends that guidance provided by experts in the field including the IXA and TTS can guide such oversight, but the support of the WHO is critical to enhance the credibility of any program. This should include worldwide distribution of the propositions to be issued, including the revision, update, ratification, and publication of updated guidelines based on the “Changsha Communiqué” as originally sanctioned by the WHO in consultation with TTS and IXA.

We propose the IXA Clinical Trial Advisory Committee (CTAC) be based upon the existing IXA Ethics Committee, which would require the expansion and addition of additional experts in the necessary fields, potentially selected from the TTS, CTAC, and IXA membership. The purpose of the IXA CTAC would be to review the safety and efficacy of a trial and provide advice to the proposed sponsor; it would also be able to advise of potential regulatory compliance requirements along with registry notification and would report to the TTS CTAC.

In addition to these approaches to improving the process of regulation, IXA suggests that one avenue could be the integration with TTS on establishing an IXA CTAC framed upon what TTS has formally established with their Clinical Trials Framework (CTF) and CTAC. IXA would aim to integrate this as part of the broader remit of the TTS, CTF and the IXA, IXA CTAC would be a standalone committee of the IXA supported by the TTS that would be available to advise and direct clinical trials of xenotransplantation, but not to be legally empowered to regulate researchers. The committee would be a credible source of information and most importantly a resource of experts to provide factual and up-to-date information for institutions and regulatory authorities considering a proposal for any xenotransplant clinical trial.

Additionally, the IXA would also propose that the xenotransplant registry would benefit from international support for personnel, data, and sample storage, along with statistical and analytic expertise, including financial support possibly from regulatory agencies for undertaking the significant tasks required for its custodianship. Alternatively, it could potentially be incorporated into a larger internationally supported and well-prescribed registry as outlined recently [48]. In summary, it is imperative that a new or significantly reinvigorated international registry be launched to more broadly and proactively collect data on worldwide xenotransplantation activities. One possible avenue could be to amalgamate the data from the existing IXA registry with the Global Observatory on Donation and Transplantation (GODT), which was established in 2008 in collaboration between the WHO, the CoE, Pan American Health Organization (PAHO), ONT, and transplantation societies. The GODT archives data on worldwide organ donation and allotransplantation and could be the platform for broader collection of all studies, leading to a comprehensive database for transparent archiving of xenotransplantation activities.

The IXA proposes that this is a timely opportunity to expand the data collected in the current registry, to include (for example) minimum data on porcine donor species and breeding, and recipient information on organ or cell transplant and type of immunosuppression used. On the other hand, it could well be served better to have the new or expanded registry hosted by an agency perceived to be neutral and based at an academic institution, if possible, as it may also allow for increased data collection that is not possible under GODT. This will require significant investment from the academic and financial perspectives.

In view of future developments and broader applications of clinical xenotransplantation, it seems logical to integrate the current xenotransplant inventory into an internationally recognized registry unless funds are provided to expand and reinvigorate the current registry with oversight provided by national and international groups. The IXA Council would elaborate on a project for the relevant data collection, joint support, for approval with TTS. TTS delegates who collaborate with WHO will take this forward for WHO sanction, including a request for ongoing support or incorporation into a larger international transplant registry [48].

## 9 | SUMMARY AND CONCLUDING REMARKS

In summary, IXA believes that the major recommendations contained in the Changsha Communiqué should be regularly revisited and renewed by international experts based on updated evidence from the xenotransplant field and beyond. This will help to ensure that the guidance documents for the field remain current with the cutting-edge technologies and advancements necessary to take xenotransplantation safely to the clinic. Preclinical studies should be sufficiently rigorous to provide confidence that a clinical trial will be safe and efficacious with a realistic chance of success but need not be so demanding that clinical trials would be impeded by very prolonged experimentation. Although preclinical studies in NHPs and decedents have been highly valuable to incrementally improve clinical xenotransplantation practices, IXA Council strongly supports current efforts to learn from “pilot” clinical xenotransplant cases: In our estimation, current evidence predicts that patients are likely to benefit significantly from a successful xenotransplant in quality and length of life and that a “successful” outcome, from the patients perspective, is possible, and in the view of some even likely. Importantly, information gained from pilot studies is highly likely to inform improved design and conduct of subsequent clinical trials for various xenograft applications. In view of the critical shortage of suitable human organs for transplantation, *IXA Council recommends that xenotransplantation should be prioritized as a global health endeavor, with the goal to design clinical xenotransplantation trial options and make them available for patients on transplant wait lists*. The focus for such trials would be on patients who either are highly likely to die on the wait list before being offered a transplant, and/or patients who currently do not get listed because they are too high-risk, or listing them is considered futile, due to long waiting times or comorbidities likely to worsen while waiting. These are, of course, base principles and require specific scrutiny for the various diseases and the organs, tissues, and cells to be transplanted and will be individually addressed in the accompanying “White Papers”.

## CONFLICTS OF INTEREST

The authors have the following conflicts of interest: Wayne J. Hawthorne, Leo Buhler, Peter J. Cowan, Raphael P. H. Meier, and Emanuele Cozzi have no conflicts of interest to declare. Richard Pierson III is a consultant to Moderna and Veloxis and receives preclinical research support from NIH (UO1 AI 153612, U19 AI 090959, and U01 AI 146248), Revivicor, eGenesis, and Tonix Pharmaceuticals. Jay Fishman reports membership on Scientific Advisory Boards for United Therapeutics, Makana, eGenesis, Elion, Jura, and Well Medical. Rita Bottino is a full-time employee of Imagine Pharma, PA, USA. Paolo Brenner and Eckhard Wolf are cofounders of XTransplant GmbH, Starnberg. Muhammad M. Mohiuddin received research support from United Therapeutics Inc.

## DATA AVAILABILITY STATEMENT

Data sharing not applicable to this article as no datasets were generated or analyzed during the current study.

## APPENDIX

On behalf of the Council of the International Xenotransplantation Association (IXA)

Previous IXA Council members:

Shaoping Deng, Sichuan Provincial People’s Hospital, Chengdu, Sichuan, China

Burcin Ekser, Transplant Research and Xenotransplantation Laboratory, Indiana University, Indiana, USA

Current IXA Council members:

Hidetaka Hara, College of Veterinary Medicine, Yunnan Agricultural University, China

Gregory Korbutt, University of Alberta, Canada

Robert Reiben, Swiss National Science Foundation project “Xeno2Cure”, Switzerland

Dr. Ik Jin Yun, Konkuk University Medical Center, South Korea
